# The Influence of Top Managers on Environmental Information Disclosure: The Moderating Effect of Company’s Environmental Performance

**DOI:** 10.3390/ijerph16071167

**Published:** 2019-04-01

**Authors:** Yuan Ma, Qiang Zhang, Qiyue Yin, Bingcheng Wang

**Affiliations:** College of Economics and Management, Shandong University of Science and Technology, Qingdao 266590, China; zhangqiang3637@163.com (Q.Z.); yinqiyue0804@gmail.com (Q.Y.); qdwbc@163.com (B.W.)

**Keywords:** environmental information disclosure, top manager, educational background, environmental performance, moderating effect

## Abstract

Abundant extant literature emphasizes the impact of board members attributes’ influence on environmental information disclosure. Considering the voluntary nature of environmental information disclosure, executives have strong managerial discretion when they make such decisions, so this article focuses on top managers’ influence on environmental information disclosure. We hypothesize that top managers’ educational background and age will affect companies’ environmental decision. The hypotheses are verified with the data from Chinese listed manufacturing companies. As the results show, a Master of Business Administration (MBA) educational background and average age of top managers positively affect environmental information disclosure, while the impact of legal educational background is negative. The company’s environmental performance plays a U-shaped moderating effect on the relationship between MBA educational background and environmental information disclosure.

## 1. Introduction

As society and the public pay more attention to environmental issues, environmental information disclosure (EID) has gradually become an important way for companies to communicate with stakeholders [1,2,3,4]. The influencing factors of voluntary EID have gained abundant attention. The extant research in this domain follows two branches. Following an out-in approach [5], scholars have studied the impact of external factors, such as institutional pressure, media supervision, stakeholders, peer competition and customers, on companies’ EID [1,6,7,8,9,10,11,12].

When facing with common sets of pressures, companies exhibit different environmental strategies [13]. Follow a strategic inside-out approach [5], some scholars further discussed the influence of board members’ preferences on EID, such as gender, age, and level of education. Gender socialization theory considers that women take the responsibility of raising children and therefore pay more attention to social and environmental issues [14,15,16]. Female board members have a positive effect on EID [3,17,18,19,20,21]. A higher level of education will affect the environmental awareness of board members, so the level of education is proportional to EID [22,23]. Board members’ age will also have an impact on EID. With the increase of age, citizens’ social responsibility is also gradually strengthened, so senior board members have a stronger sense of environment [24,25,26].

EID is a part of company strategy. In addition to the impact of the above board characteristics, the resource base of the company will also affect the EID [27,28,29,30,31,32,33,34,35]. Environmental performance is an important factor. Two points of view have been formed by existing research. The theory of information disclosure holds that EID is a tool for companies to reduce information asymmetry, so companies with good environmental performance are more willing to release environmental information [27,28]. Legitimacy theory states that EID is a tool for companies to carry out image management, and companies with poor environmental performance could beautify the corporate image by actively publishing environmental information [4,31]. Empirical researches in this domain result in mixed results. For example, some analyses show a positive relationship between environmental performance and EID [29,30], while others provide opposite evidence [32,33,34].

Although extant literature provides plenty of explanations for the companies’ heterogeneous EID behavior, the focus of academic attention remains on the impact of the board, ignoring the impact of executives. EID is not mandatory, companies cannot accurately calculate the costs and benefits associated with it, so the executives’ perception and their personal judgment affect the decision of the company to a large extent [13,36]. Executives will make limited rational choices based on the specific circumstances of the business [37]. In the study of EID, there is still a lack of integration of executive characteristics with the specific situation of the company [23]. This study revolves around this research gap. There are two issues of concern to us: (1) Can executive characteristics affect the EID decision of a company? (2) When executives make environmental decisions, can the specific situation of the firm affect the outcome of the decision? We posit that whether to proceed EID is a prudent decision made by decision makers based on the specific situation of the company and the professional judgment of individuals. A strategic inside-out approach is followed in this paper. External factors, such as stakeholder pressure, regulation, are set as control variables and aren’t put emphasis on.

The research sample of this paper is China A-Share listed companies. As the world’s largest developing country, China has consumed a lot of natural resources and emitted huge amounts of pollutants while sustaining rapid economic growth. Therefore environmental issues are very prominent [21,38,39]. Over the past decade, Chinese government has invested a great deal of energy in environmental protection. In 2014, the government revised the Environmental Protection Law, strengthened the responsibility of corporate pollution prevention and control, increased legal sanctions for environmental violations, and also made provisions on the disclosure of environmental information, public participation, environmental supervision and so on [21]. In the Fifth Plenary Session of the 18th Communist Party of China Central Committee in 2015, “beautiful China” was included in the 13th Five-Year plan as a basic national strategy, and was planned to be basically achieved in 2035. Although the central Government has attached great importance to environmental protection and pollution control, as a transition economy, there is still a long way to go before the growth mode of high pollution and energy consumption could be converted to sustainable development. Compared with developed countries such as the United States and Australia, China has a relatively low level of education in sustainable development [40], the environmental impact of social groups is relatively weak [39], and there is still a lax enforcement in specific implementation process [38]. Therefore, the enthusiasm of company environmental management is relatively low. Specific to the EID of listed companies, in 2006 and 2007 respectively, two major domestic stock exchanges (Shenzhen Stock Exchange and Shanghai Stock Exchange) promulgated relevant approaches to guide listed companies to voluntarily disclose environmental information, and followed by the revision of specific rules, but so far, EID is still an act of voluntary disclosure, heavy pollution industries are exception. Considering China’s particularity in environmental regulation and supervision, compared to their Western counterparts, Chinese companies do not pay enough attention to EID. Therefore, it is very necessary to study the EID behavior of Chinese companies. Research results in such situations are still scarce [16,20,21] and our research could provide more evidence.

This article makes several contributions. First, the influencing factor of EID is analyzed based on the characteristics of top managers in this research. Studies have focused on the impact of board members, although a small amount of literature has studied the impact of executive characteristics on corporate strategy [41], research linked to EID remains limited. The Upper Echelons theory believes that executives play a vital role in the process of strategic choice and strategy implementation. Therefore, studying the influencing factor of EID from the standpoint of top management team is conducive. Studying the EID of the company from the perspective of the executive team will help to deepen the understanding of the company’s environmental strategy [42]. Second, this paper mainly focuses on the impact of the educational background of executives on EID, and the research on the impact of educational background on information disclosure is also scarce. Our findings suggest that executives’ perceptions of environmental issues are related to their educational background. This provides evidence for the effectiveness of sustainable education and environmental education. Third, the environmental performance of a company has been added to our research model as a moderating variable. The results show that professional managers will make environmental decisions carefully according to the actual situation of companies. This provides evidence for enhanced environmental regulation and information disclosure audits. The remainder content is organized as follows. The most relevant theories and the hypotheses are given in Section 2. The variables and samples are described in Section 3, then the analysis results and discussions in Section 4. Conclusions and implications are presented in the last section.

## 2. Hypotheses Development

### 2.1. Top Managers’ Traits and EID

Upper Echelons theory holds that many decisions of companies have multi-objective, non-quantitative and fuzzy characteristics. Executives can only make their decisions within the bounds of rationality [37]. The particular experience and personal characteristics of executives will affect their personal cognitive styles, which in turn leads top managers’ different opinions and choices on companies’ decision issues.

With the deterioration of natural environment and the public’s desire for sustainable development, the impact of natural resources (natural environment) on companies is gradually increasing. In this context, Hart [43] proposed and improved the Natural-Resource-Based theory. The theory holds that the rapid growth of population, the massive consumption of resources, the discharge of hazardous, and harmful wastes have caused great damage to the nature, which is far beyond the self-repairing ability of the earth. Companies, who consume huge resources and produce a large number of environmental pollutants, cannot overlook this changing business environment. Therefore natural environment has become an important factor to be considered in business operations. EID is one of the initiatives that companies take when dealing with environmental problems. Because it is difficult for companies to accurately measure the corresponding costs and benefits of EID, top managers have a great managerial discretion. Executives always make decisions according to their judgments. Top managers’ characteristics, especially their background and experience, will form their perceptions about environmental problems [13], which in turn will affect the executives’ decisions to disclose environmental information or not.

Combined with the professional characteristics of executives, this study focuses on the impact of educational background on EID. The influence of executives’ Master of Business Administration (MBA) educational background and legal educational background are proposed. We choose these two types of educational backgrounds because they are often used in the domain of corporate strategy and financial information disclosure [8,17,26]. Besides these, we put forward a competitive hypothesis about the influence of executives’ age which is contrary to prior research.

#### 2.1.1. Top Managers’ MBA Educational Background

Studies show that executives with MBA educational background have higher preferences in such behaviors as capital investment, liability, and diversified mergers and acquisitions [26]. This is because such executives have received more professional training in business management, have higher management skills, and have better assessment techniques when facing with complex problems [44]. Such executives are more receptive to new things and more responsive to business environment change [45].

Facing with the increasing environmental concerns of the government and the public, EID is a common strategy used in environmental management. It can convey companies’ corporate social responsibilities and environmental responses, promote corporate image and reputation [22,30,46], enhance corporate legitimacy [47], and improve corporate profitability [48,49]. In addition, EID can also influence investors in the capital market, reduce companies’ financing cost [50], increase cash flow [34], and enhance market value [51]. It is an opportunity that can be utilized in business operations [52]. Based on the above analysis, we believe that executives with MBA educational background are more likely to view environmental problems as an external opportunity, and therefore use EID as communication tool to grasp this external opportunity. The following hypothesis is postulated:

**Hypothesis 1** **(H1):** *MBA degree of top managers will increase their propensity to disclose environmental information, ceteris paribus.*

#### 2.1.2. Top Managers’ Legal Educational Background

People with legal educational experience tend to behave conservatively because of their professional norms [53]. Legal professional norms tell them to keep clients’ secrets and minimize risks [8]. When making decisions, they usually adopt pessimistic attitude and reduce future expectations, and are very sensitive to possible litigation issues [17].

Executives with legal degrees present a unique style in their decision-making [13]. When they become top managers, they take on a large responsibility for companies’ decision. To reduce operational risk, they prefer to remain the current status and will be more cautious [54]. Prior research shows that top managers with legal educational background will reduce investment when facing uncertainty [17] and guide down earning forecast [55].

As far as EID is concerned, some studies suggest that such information disclosure will increase companies’ risk compared to other information disclosure [56]. Firstly, external stakeholders, such as government and environmental organizations, may use its environmental information to initiate litigation or boycott against the focal company [57,58]; secondly, such disclosure may leak key information and technology and let its’ competitors know its strategic intentions [59]. Improper EID will increase the risk of the company. Executives with legal educational background always tend to be risk-aversion [13,53].

Combined with the above analysis, it is reasonable to believe that executives with legal educational background will regard environmental problems as threats and therefore take a conservative attitude. Therefore we propose the following hypothesis:

**Hypothesis 2** **(H2):** *Legal educational background of top managers will decrease the propensity to disclose environmental information, ceteris paribus.*

#### 2.1.3. Top Managers’ Age

Executives’ age affects their values, cognitive styles and decision [37]. People born in different ages have different growth experiences and thus different attitudes towards the same issue. For example, executives born before World War II have conservative values because they have experienced war and hunger [60]. Empirical studies have shown that senior executives have lower preferences in terms of financial leverage, and their decision-making styles tends to be conservative [61]. In addition, as years going by, people are more incline to maintain the current state and balance, and thus reject new ideas and practices [62]. Compared to senior ones, junior executives are more energetic and creative, more incline to accept new ideas and new management methods [63,64]. They always pursue innovation proactively [13]. Therefore, junior executives are more receptive to the rules of EID [65,66]. Although existing studies have proposed that there is a positive relationship between executives’ age and EID, we propose a competitive hypothesis that executives tend to be conservative with the increase of age, and therefore regard environmental issues as a threat and will be more passive when they make EID decision.

**Hypothesis 3** **(H3):** *The propensity to disclose environmental information inclines as the top managers’ average age increases, ceteris paribus.*

### 2.2. The Moderating Effect of Environmental Performance

From the perspective of voluntary disclosure, some studies believe that EID is an important way for companies to reduce information asymmetry with their stakeholders, so companies with good environmental performance will release environmental information proactively [47]. From the perspective of legitimacy theory, studies suggest that EID is a tool for companies to maintain their legitimacy, therefore companies with poor environmental performance will more incline to release information to wash themselves green [52]. The empirical research provides supports for the above theories respectively [27,28,29,30,31,32,33,34]. Considering the findings mentioned above, we argue that companies’ environmental performance plays an important role when executives make their EID decisions. When companies’ environmental performance is better, executives will be more inclined to release environmental information to reduce information asymmetry and publicize a good company image; when companies’ environmental performance is poor, executives will also be inclined to issue environmental information reports to improve company image through this way. Therefore, environmental performance plays a U-shaped moderating effect in the relationship between top managers’ attributes and EID. Subsequently, the following hypotheses are proposed:

**Hypothesis 4a** **(H4a):** *Environmental performance plays a U-shaped moderating effect on the relationship between top managers’ MBA educational background and EID.*

**Hypothesis 4b** **(H4b):** *Environmental performance plays a U-shaped moderating effect on the relationship between top managers’ law educational background and EID.*

**Hypothesis 4c** **(H4c):** *Environmental performance plays a U-shaped moderating effect on the relationship between the top managers’ average age and EID.*

**Figure 1:**
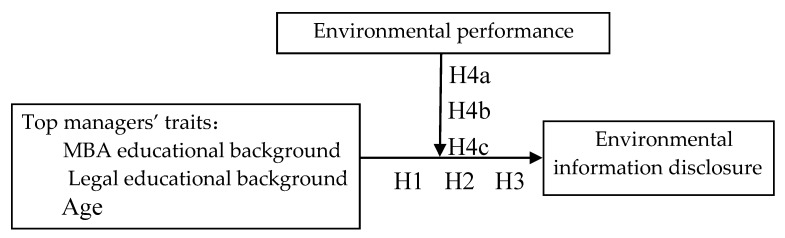
Research framework.

Research frame is shown in Figure 1.

## 3. Research Design

### 3.1. Research Variables

#### 3.1.1. Independent Variables

MBA educational background: take the proportion of the number of top managers with MBA degree to the size of the top management team. 

Legal educational background: take the proportion of the number of top managers with legal educational background to the size of the top management team.

Age: it is measured by the natural logarithm of top managers’ average age.

#### 3.1.2. Dependent Variable

EID: It takes the value of “1” if the company discloses its environmental information; “0” otherwise. Although a continuous variable, named environmental disclosure index, is often used to measure companies’ disclosure content in prior research [16,20,21], what we care about is whether the company discloses environmental information or not, so a binary variable is used in our study.

#### 3.1.3. Moderating Variable

Drawing on existing research [4], the number of green technology patents of the company is taken as a proxy variable. By searching for company patent data from the Baiten patent website manually and setting keywords such as “green” or “sustainable development” or “energy saving” or “emission reduction” in the search engine to filter the patent data, we finally obtained companies’ green technology patent data. Natural logarithm is taken after the number of patents plus 1.

#### 3.1.4. Control Variables

Considering prior research from corporate governance theory and regulation theory, the following control variables are added:

Listing location: there are two stock exchanges in China, Shanghai Stock Exchange and Shenzhen Stock Exchange. The two stock exchanges have different requirements and regulatory methods for information disclosure of listed companies, which may affect the information disclosure decisions of listed companies.

Company size: the larger the company, the higher the public’s attention, so company size may affect EID positively [35]. It is measured by the natural logarithm of the value of the year-end book assets (million yuan).

Proportion of shares held by the largest shareholder: some studies propose that the degree of dispersion of shareholders determines whether companies need to disclose relevant information to reduce information asymmetry [3]. As a result, the higher the shareholding ratio of the first largest shareholder, the less likely the company to disclose environmental information. It is calculated as the total number of shares held by the largest shareholder divided by total number of shares released.

State ownership: Institutional pressure is an important factor that enables companies to disclose environmental information [1,2,4,5]. In China, where the government has more regulatory powers over state-owned companies, so, state-owned companies are under stronger institutional pressure and are more likely to release environmental information [21]. If a company’s largest shareholder is a state-owned or a state-controlled company, the value is 1, otherwise, it is 0.

Board size: Board composition is also an important factor that influences companies’ EID initiatives. The larger the board size, the higher the possibility of information disclosure [67,68]. It is measured by the natural logarithm of the number of board members.

Chief Executive Officer (CEO) duality: CEO duality means that the Chairman of the company is also the CEO. Due to the concentration of power, when the chairman is the same person as the general manager, he (she) will pay more attention to personal issues and ignore the interests of shareholders. On the contrary, the separation of two roles is conducive to improving the quality of regulation and transparency of information [69]. Some studies have found a negative relationship between CEO duality and environmental information disclosure. When the Chairman and general manager is the same person, the value of this variable is 1, otherwise it is 0.

See Appendix A for a description of the variables.

### 3.2. Research Sample

The manufacturing industry consumes a large amount of energy and natural resources and emits a huge amount of pollutants, so it is often selected as a research sample in the domain of environmental management. The sample selected in this study is listed manufacturing companies in Shanghai Stock Exchange and Shenzhen Stock Exchange in China. We obtain the industry classification of A-share listing companies and retain manufacturing companies. EID of heavy polluting industries is mandatory in China, executives do not have managerial discretion, so executive characteristics do not affect EID. Considering this reason, heavy polluting industries, such as papermaking industry, chemical industry, and pharmaceutical industry, are excluded. The time interval is set from 2015 to 2017, considering a national strategy to build a “beautiful China” has been established since 2015. This results in having 943 companies in 2015, 1038 in 2016, 1235 in 2017. The information of environmental performance is gotten from Baiteng Patent database. The information of other variables are selected from the CSMAR Database, a widely used database for listed companies in China. In order to overcome the influence of outliers on the research conclusions, Winsorize processing is carried out on the main continuous variables at 1% and 99% quantiles. Excluding missing data, the final sample includes 2329 company-year observations.

## 4. Analysis Result

### 4.1. Descriptive Statistics and Correlation

Descriptive results and correlation coefficients of the above main variables are shown in Table 1. In the research samples, 40.9% of the companies come from Shanghai Stock Exchange, 39.4% are state-owned. 28% of the sample companies release environmental information. Since this study only considers voluntary EID and does not include heavy pollution industries, the proportion of information disclosure is lower than that of other studies [35]. The average proportion of members with MBA educational background is 26.3%, which is lower than that of American listed companies [55]. The ratio of legal educational background is 5.7% on average, which is relatively lower than that of American listed companies [55]. Top managers’ average age is 48.57. Average number of board size is 9. In terms of the correlation coefficients between variables, the correlation coefficient between state ownership and the proportion of shares held by the largest shareholder is higher than 0.5. In order to reduce the multicollinearity problem, the proportion of shares held by the largest shareholder is retained in the model and state ownership is used in the robustness test. The correlation coefficients between other variables are low and not significant, indicating that multicollinearity problem could be ignored in this study.

### 4.2. Regression Model and Results

As the dependent variable is binary, the above hypotheses are verified using logistic regression. Let $X_{i}(i=1,2,3)$ be the independent variable in the model, where $X_{1}$ denotes the proportion of top managers with MBA educational background; $X_{2}$ denotes the proportion of top managers with legal educational background; $X_{3}$ denotes the average age of top management team; Y denotes EID, if the company release environmental information Y=1, otherwise, Y=0; CTR denotes control variables; *P* denotes the response probability. The regression model is as follows:

(1) $$\ln\frac{P(Y=1)}{P(Y=0)}=\alpha +\beta_{1}X_{1}+\beta_{2}X_{2}+\beta_{3}X_{3}+{\sum \beta_{i}CTR}_{i}$$

The probability of event occurrence can be obtained by transformation:

(2) $$P(Y=1)=\frac{e^{\left(\alpha +\beta_{1}X_{1}+\beta_{2}X_{2}+\beta_{3}X_{3}+{\sum \beta_{i}CTR}_{i}\right)}}{1+e^{\left(\alpha +\beta_{1}X_{1}+\beta_{2}X_{2}+\beta_{3}X_{3}+\sum \beta_{i}CTR_{i}\right)}}$$

Further the occurrence ratio of the event can be obtained:

(3) $$odd(Y=1)=e^{\alpha +\beta_{1}X_{1}+\beta_{2}X_{2}+\beta_{3}X_{3}+\sum \beta_{i}CTR_{i}}$$

When the estimated value of parameter β in the regression model (1) is positive, it indicates that the occurrence ratio of EID will be increased with the per unit increase of the independent variable.

“EID” is put into the dependent variable, and the aforementioned control variables and independent variables are put into the covariate using logistic regression by SPSS (SPSS Inc., Chicago, IL, USA). The classification threshold is set to 0.5 and the maximum number of iterations is set to 20. After five iterations of the system, the results are effectively converged, as shown in Table 2. The control variables are included in Model 1, and the results show that listing location, company size, board size and proportion of shares held by the largest shareholder have a positive impact on EID. Three independent variables are added to Model 2, and the coefficients of Cox & Snell R^2^ and Nagelkerke R^2^ in the this model are significantly increased, indicating that the added independent variables have explanatory power for dependent variables. The influence of MBA educational background on EID is positive and significant. So Hypothesis 1 is supported. The influence of legal background on EID is negative and significant. Hypothesis 2 is also verified. The coefficient of age is positive and significant, which is contrary to our expectation. Hypothesis 3 fails to be supported.

The non-linear moderating effects in the study are tested referring to Bhuian et al. [70]. The MBA educational background, environmental performance, and squared environmental performance are standardized, and the product of MBA educational background and squared environmental performance is formed. The above variables are put into Model 3. The results are shown in Table 3. The Cox & Snell R^2^ and Nagelkerke R^2^ coefficients of the entire model are further improved, indicating that the prediction effect of the new model on the dependent variable is improved. The product of MBA and the square of environmental performance has a negative and significant coefficient, indicating that the inverted U-shaped moderating effect exists. So H4a is verified. Repeat the above steps to test H4b and H4c. The results are depicted by Model 4 and Model 5 respectively. Since the coefficient corresponding to the product of legal background and the square of environmental performance is non-significant, H4b fails to be supported. H4c fails be tested either.

In order to test the robustness of the above models, we use an alternative control variable, state ownership, to repeat the above models. The coefficients and significance levels are similar to the above results.

### 4.3. Discussions

#### 4.3.1. Control Variables

Listing location can affect the company’s EID. Companies listed in Shanghai Stock Exchange are more likely to disclose environmental information. This may be due to the special disclosure principles issued by Stock Exchange. For example, Shanghai Stock Exchange started to evaluate the disclosed information of listed companies since the year of 2016, revised the information disclosure principles in the year of 2017, and will increase the support strength for proactive environmental responsible companies in the future. These measures promote the enthusiasm of EID of listed companies to a certain extent.

Company size has a positive impact on EID, which is consistent with the conclusion of McGuinness et al. and Luo et al. [16,71]. This is because large companies are more likely to attract attention from the media and the public. The proportion of shares held by the largest shareholder is positively related to EID. This is contrary to what we expected. A possible explanation for this result is that shareholders with a high shareholding ratio are more concerned with the corporate image and reputation [72]. Board size has a positive impact on EID, which is consistent with Barakat et al. [67]. The impact of CEO duality is negative but insignificant, which is consistent with Lasasio and Cucari [68].

#### 4.3.2. Independent Variables and Moderating Variable

Executives’ MBA educational background has a positive impact on EID. We attribute this to the special human capital of such executives. During the MBA education, students will be exposed to a large number of strategic management, business ethics and other aspects of knowledge and education. Systematic thinking training allows them to be keenly aware of changes in the external environment and to respond appropriately. After year 2015, Chinese government has stepped up environmental regulation and gradually implemented environmental supervision in provinces, ordering companies with substandard environmental protection to shut down and rectify. Environmental responsibility has become an important index to evaluate company performance. In the face of these external changes, executives with MBAs are keenly aware of the potential benefits that EID could bring to the company.

The legal education background of executives negatively affects the decision of EID. This is also due to the special human capital of such executives. Such executives are familiar with the law and are well aware of the harm caused by improper disclosure of information, such as potential litigation risk and disclosure risk. Since environmental information is voluntarily disclosed, they would rather not disclose it than face possible risks.

Young executives are more likely to adapt to volatile environments and be able to respond quickly to the external environment. Based on this reasoning, we propose Hypothesis 3, in which the age of the executive is inversely proportional to the disclosure of information. But the empirical result is contrary to what we envision. Previous studies have shown that citizens’ sense of social responsibility is gradually increasing as they get aged, so that such executives show a higher preference for EID [9,26]. After consideration, we think that age is a bio-demographic feature, and its correlation with the responsibilities of executives is not high. So the impact of this type of feature on executive decisions is more about complying with universal interpretation of ethicality theories.

By comparing Hypothesis 1, Hypothesis 2 and Hypothesis 3, we further infer that the background of MBA education and the background of legal education are task-related attributes, and that professional education increases the human capital of executives, enables them to consider more about their job needs when making decisions. Age belongs to bio-demographic attribute, and other universal theories need to be considered when predicting the impact of such characteristics on executive decision-making.

Environmental performance has a U-shaped moderating effect on the relationship between MBA educational background and EID, while this moderating effect doesn’t exist in the other two contexts. This conclusion reinforces our aforementioned inference. Because compared with the legal background, executives with MBA degrees have received professional training in business and have the ability of overall thinking, the internal and external environment are the important aspects that they need to consider when making decisions, so the environmental performance of companies will affect their environmental decision-making. EID is a tool available to businesses with particularly good (or particularly poor) environmental performance [50,52].

## 5. Conclusions

### 5.1. Key Findings

Although EID is voluntary, it is never decided randomly since this is a prudent decision made by executives in conjunction with their own perceptions and the realities of the business. Using a large sample of Chinese A-shared listed manufacturing companies, our research get the following findings. Firstly, our findings show that top managers’ MBA educational background has a positive relationship with EID, and companies’ environmental performance plays a U-shaped moderating effect. When facing environmental problems, MBA education executives are better at making flexible judgments. Secondly, top managers’ legal educational background has a negative impact on EID, and this effect has nothing to do with firm’s environmental performance. The legal education background makes such executives delay the risk. They prefer to do less rather than make mistakes. Thirdly, top managers’ age also has a positive impact on EID, which is accordance with prior research and can be explained by universal interpretation of ethicality theories. The positive impact of executive’s age is also irrelevant to corporate factors.

### 5.2. Implications

The conclusions of this paper can provide enlightenment for academia, policy makers, information regulatory departments and educational industry.

Firstly, our result shows that top managers’ characteristics play a relatively important role in companies’ environmental decision. It gives complementary evidence to Bamber et al. [55] who discuss top managers’ impact on companies’ disclosure choice.

Secondly, EID is a part of company’s environmental strategy, in order to induce companies to take on environmental responsibility actively, policy makers can adopt diversified market-oriented regulatory measures to promote the change of business environment actively, and enhance business managers’ opportunity perception further.

Thirdly, the research results show that the disclosure decision is influenced by the actual operation of the firm, and it is the professional judgment made by top managers by considering the environmental performance of the company. Companies with particularly good or poor environmental performance tend to disclose environmental information actively, which indicates that EID is a management tool for companies to take advantage of external opportunities [73,74]. To disclose environmental information whether or not is a decision made by executives based on their professional judgment. It should be emphasized that there is evidence showing that MBAs’ cheating level in self-reported exams is higher than other majors, and those students who are dishonest in their studies are also likely to be dishonest in their work [75]. Executives are likely to disclose environmental information selectively to both attract stakeholders and meet their own needs [74,76]. Therefore, environmental information auditing is very necessary. Financing audit which is compatible with environmental information assessment in the capital market is helpful. It can improve the enthusiasm of companies’ EID and increase companies’ attention to environmental problems. Besides this, more detailed information dissemination guidelines, authorized third parties to carry out information quality assessment and periodic evaluation results announcement and some other measures can also be a constraint on the quality of company’s EID, to prevent companies with poor environmental performance to take advantage of this opportunity.

Finally, professional education can influence the cognitive and professional judgment of executives. The MBA program has courses related to business ethics and sustainability to help students build the concept of sustainability while other majors rarely involve such courses. This has resulted in a different impact of educational background on executive decision-making. The issue of sustainable development is becoming more and more urgent, so it is very necessary to popularize sustainable education in universities [40]. This will raise environmental awareness among the entire population, as well as provide more talent to meet the economic transformation.

### 5.3. Limitations and Future Research Directions

In previous studies, the most discussed board attributes are gender, age and level of education. Combining with the professional needs of executives, this paper focuses on the impact of MBA educational background, legal background and age on executives’ environmental decision. Since the domain of the Upper Echelons theory mainly focuses on company strategy and pays little attention to EID, this research has made a preliminary exploration. It leads to the limitation of this research that the characteristics of the executives involved are relative few. In addition, this study is placed in developing countries. Because the same type of research is still scarce, there is a lack of comparison of the research conclusions. This may affect the universality of the findings of this study.

Companies cannot make accurate trade-offs between environmental information decisions from the point of view of cost and benefit, and executives have discretion in decision-making. There are many similar decision-making issues, such as green innovation decisions in companies, so the follow-up research can spread to other situations. In addition, besides the educational background mentioned in this article, other attributes could also be included, such as executives’ functional career tracks. More importantly, future research could consider the interplay of task-related attribute and bio-demographic attribute, which would help uncover companies’ environmental decisions.

## Figures and Tables

**Table 1:** The descriptive statistics and their correlation coefficients.

	Loc	Own	Dua	Csize	Prop	Bsize	EP	MBA	Legal	Age	EID
Loc	1										
Own	0.096 **	1									
Dua	−0.069 *	0.039	1								
Csize	0.251 **	0.161 **	0.032	1							
Prop	0.071 *	0.582 **	0.049	0.173 **	1						
Bsize	0.026 *	0.014 *	0.037	0.161 *	0.046	1					
EP	−0.010	0.022	−0.010	−0.016	0.038	0.043	1				
MBA	−0.249 **	−0.006	0.068	−0.052	0.005	0.031	0.009	1			
Legal	0.049	−0.038	−0.037	0.020	0.040	0.017	−0.025	0.057	1		
Age	0.051	0.052	0.084 *	0.032	0.025	0.022	0.077 *	−0.008	−0.040	1	
EID	0.242 **	0.067	0.012	0.351 **	0.117 **	0.112 **	0.017	0.032 **	−0.059 **	0.080 *	1
Mean	0.350	0.596	0.890	3.720	0.312	9.012	1.412	0.263	0.057	3.873	0.280
S.D.	0.478	0.321	0.311	0.527	0.141	2.374	1.412	0.200	0.516	0.141	0.449

** *p* < 0.05, * *p* < 0.1. S.D. = standard deviation; Loc = listing location; Own = state ownership; Dua = chief executive officer duality; Csize = company size; Prop = proportion of shares held by the largest shareholder; Bsize = board size; EP = environmental performance; MBA = the proportion of MBA; Legal = the proportion of legal background; Age = the average age of top managers; EID = Environmental information disclosure.

**Table 2:** Regression results.

Variables	Model 1			Model 2		
	B	Wald	Exp(B)	B	Wald	Exp(B)
Intercept	−7.401	91.962	0.001	−11.778	20.531	0.000
Loc	0.823 ***	61.188	2.277	0.860 **	60.669	2.364
Dua	−0.073	0.070	0.930	−0.033	0.014	0.968
Csize	1.415 ***	68.257	4.117	1.412 ***	64.787	4.106
Bsize	0.612 ***	23.175	1.844	0.609 ***	22.522	1.838
Prop	1.860 **	14.466	6.425	1.923 ***	15.061	6.839
MBA				1.592 **	23.668	4.912
Legal				−0.205 **	16.204	0.815
Age				1.102 **	8.526	3.012
Chi-square	354.085 ***			371.079 ***		
−2 log likelihood	2370.495 ***			2338.016 ***		
Cox & Snell R^2^	0.142			0.153		
Nagelkerke R^2^	0.205			0.221		

*** *p* < 0.01, ** *p* < 0.05. Loc = listing location; Dua = chief executive officer duality; Csize = company size; Bsize = board size; Prop = proportion of shares held by the largest shareholder; MBA = the proportion of MBA; Legal = the proportion of legal bacakground; Age = the average age of top managers; the dependent variable is EID.

**Table 3:** The results of moderating effect.

Variables	Model 3			Model 4			Model 5		
	B	Wald	Exp(B)	B	Wald	Exp(B)	B	Wald	Exp(B)
Intercept	−11.583	56.775	0.000	−11.661	56.824	0.000	−11.691	57.547	0.000
Loc	0.860 ***	60.365	2.362	0.862 ***	60.787	2.367	0.852 ***	58.984	2.343
Dua	−0.035	0.016	0.966	−0.038	0.025	0.963	−0.042	0.023	0.959
Csize	1.414 ***	64.398	4.114	1.412 ***	63.926	4.105	1.418 ***	65.298	4.131
Bsize	0.610 ***	22.471	1.840	0.610 ***	22.368	1.840	0.613 ***	23.013	1.846
Prop	1.926 ***	15.094	6.859	1.925 ***	15.075	6.853	1.926 ***	15.079	6.860
MBA	1.180 ***	18.108	3.254	1.591 ***	23.186	4.908	1.665 ***	25.694	5.285
Legal	−0.209 ***	19.241	0.811	−0.201 ***	16.762	0.818	−0.203 ***	18.562	0.816
Age	1.110 **	8.550	3.304	1.108	7.966	3.028	1.136 **	8.918	3.114
EP	0.045	1.016	1.046	0.033	1.539	1.034	0.024	1.260	1.024
EP^2^	−0.157	2.163	0.855	−0.146	2.251	0.864	−0.114	2.259	0.892
MBA × EP^2^	−0.128 **	4.385	0.880						
Legal × EP^2^				0.119	0.171	0.887			
Age × EP^2^							0.059	0.433	1.061
Chi-square	371.196 ***			371.332 ***			372.223 ***		
−2 log likelihood	2337.889			2337.762			2336.871		
Cox & Snell R^2^	0.157			0.155			0.154		
Nagelkerke R^2^	0.226			0.222			0.222		

*** *p* < 0.01, ** *p* < 0.05. Loc = listing location; Dua = chief executive officer duality; Csize = company size; Bsize = board size; Prop = proportion of shares held by the largest shareholder; MBA = the proportion of MBA; Legal = the proportion of law; Age = the average age of top managers; EP = environmental performance; EP^2^ = the square of environmental performance; MBA × EP^2^ = MBA × the square of environmental performance; Legal × EP^2^ = Legal × the square of environmental performance; Age × EP^2^ = Age × the square of environmental governance; the dependent variable is EID.

## Appendix A

**Variables**	**Explanations**
EID	=1, if the company discloses environmental information; =0 otherwise.
MBA education background	The number of top managers with MBA educational background divided by the number of the top management team.
Law education background	The number of top managers with legal educational background divided by the number of the top management team.
Age	Logarithm based on the average age of top management team.
Environmental performance	Logarithm is taken after the number of green technology patents plus 1
Listing location	=1, if listed on Shanghai Stock Exchange; =0, otherwise.
Company size	Takes the logarithm of the value of the year-end book assets (million yuan)
Proportion of the largest shareholder	The total number of shares held by the largest shareholder divided by total number of shares released.
State ownership	=1, if the largest shareholder is a state-owned company; =0, otherwise.
Board size	Logarithm of board member numbers
CEO duality	=1, if the Chairman of the company is also the CEO; =0, otherwise.
